# Cancer Stem Cells of Diffuse Large B Cell Lymphoma Are Not Enriched in the CD45^+^CD19^-^ cells but in the ALDH^high^ Cells

**DOI:** 10.7150/jca.35000

**Published:** 2020-01-01

**Authors:** Shupeng Song, Yongguo Li, Kaili Zhang, Xi Zhang, Yanxin Huang, Mingyan Xu, Shuangxing Li, Xue Guan, Tao Yang, Zhiyu Liu, Jie Jiang, Yunping Luo, Yinghua Lan

**Affiliations:** 1 Department of Infectious Disease, The First Affiliated Hospital of Harbin Medical University, Harbin, 150000, Hei Longjiang, China.; 2 Animal experimental center, The Second Affiliated Hospital of Harbin Medical University, Harbin, 150000, Hei Longjiang, China.; 3 Instrument Center, Harbin Veterinary Research Institute, Chinese Academy of Agricultural Sciences, Harbin, 150000, Hei Longjiang, China.; 4 Central Laboratory, The First Affiliated Hospital of Harbin Medical University, Harbin, 150000, Hei Longjiang, China.; 5 Department of Pathology, The First Affiliated Hospital of Harbin Medical University, Harbin, 150000, Hei Longjiang, China.; 6 Department of Immunology, Institute of Basic Medical Science, Chinese Academy of Medical Science and Peking Union Medical College, Beijing,10005, China.

**Keywords:** CD45^+^CD19^-^, ALDH activity, CSCs markers, diffuse large B cell lymphoma

## Abstract

Although the existence of cancer stem cells (CSCs) has been suggested in diffuse large B cell lymphoma (DLBCL), there is still no definitive marker. CD45^+^CD19^-^ has been regarded as a potential marker of CSCs in mantle cell lymphoma (MCL). So, we explored the role of CD45^+^CD19^-^ in DLBCL. However, both CD45^+^CD19^-^ cells and CD45^+^CD19^+^ cells did not generate tumors until more than 100,000 cells were inoculated in NOD/SCID mice, even CD45^+^CD19^+^ cells generated more and larger tumors, as well as the soft agar colony formation in vitro; The aldehyde dehydrogenase (ALDH) activity was also identified in this study. Only 1,500 ALDH^high^ cells were enough to generate tumors in mice while the same number of ALDH^-^ cells were not. Moreover, both groups formed tumors when more cells were inoculated, but ALDH^high^ cells formed more and larger tumors. The similar result was obtained in vitro clonogenicity experiments. OCT4, SOX2, Nanog, and ABCG2 genes did not show any difference in CD45^+^CD19^+^, CD45^+^CD19^-^, ALDH^high^ and ALDH^-^ cells. Taken together, CSCs are not enriched in the CD45^+^CD19^-^ cells but in the ALDH^high^ cells in DLBCL cell lines.

## Introduction

DLBCL is the most common lymphoid neoplasms worldwide, accounting for about 40% of non-Hodgkin's lymphoma (NHL) cases in different geographic regions 1. Roughly 10% of patients with DLBCL are Epstein-Barr virus (EBV) positive, a higher proportion in the elderly and immunocompromised patients 2. Resting B cells can be transformed into lymphoblastoid cell line (LCL) by EBV in vitro. LCL-like cells are observed in vivo and typical in EBV-associated lymphoma patients with immunodeficiency 3. Therefore, LCL provides an important lymphoma model in vitro 4. However, the pathogenesis of DLBCL is still obscure at present. In addition, there is a risk of relapse or refractory up to 40% with chemotherapy 5.

An increasing number of evidences show that CSCs exist in many cancers 6-11. CSCs hypothesis indicates that the reason for tumorigenesis, metastasis and recurrence is related to CSCs in tumors. Recently, a report showed that the existence of “side population” (SP) cells suggested the possibility of CSCs in DLBCL 12 although there were no distinct markers for DLBCL CSCs. CD45^+^CD19^-^ has been identified as a potential marker of CSCs in MCL 13-15. CD45^+^CD19^-^ cells isolated from MCL primary patient cells generated tumors in all mice. On the contrary, mice inoculated with CD45^+^CD19^+^ cells did not generate any tumors 13. In addition, CD45^+^CD19^-^ cells were associated with the chemotherapy resistance and clinical outcomes of patients with MCL 14, 15. According to the previous study in MCL, we explored to identify whether CD45^+^CD19^-^ can be a marker of CSCs in DLBCL.

Cellular activities, such as the ALDH enzymatic activity and the above-mentioned SP cells have been used to identify CSCs populations as well. ALDH is an enzyme in the cytoplasm that facilitates the oxidation of intracellular aldehydes into acids. It is expressed in various stem/progenitor cells. Compared with cell surface markers, the cellular intrinsic functional property ALDH activity is more generally accepted in different types of tumor, such as leukemia, liver, lung, breast, colon and head and neck cancers 16-21. However, whether ALDH high activity is suitable as a marker to enrich DLBCL CSCs has not been reported, although the previous studies showed high expression of ALDH1A1, an isoform of ALDH, mediated chemo-resistance and associated with worse prognosis in DLBCL by immunohistochemistry 22-24. So ALDH^high^ activity cells were sorted using Aldefluor assay kit by flow cytometry and explored the possibility as a marker of DLBCL CSCs in our study.

## Materials and Methods

### Cell lines and cultures

An EBV-transformed LCL was established. The EBV-transformed marmoset cell line B95-8 was purchased from Kunming Cell Bank of Chinese Academy of Sciences. It was grown to confluency, and infectious culture supernatants were collected and stored at -80℃ before use. A healthy donor samples of peripheral blood were separated by Ficoll-Hypaque gradient centrifugation to acquire peripheral blood mononuclear cells (PBMC). Six million PBMCs of 3 ml complete medium was added to 3 ml of B95-8 supernatant in a 25 cm^2^ culture flask. Clusters of cells were observed by a light microscopy about a week later and became larger over time. The cell culture medium was changed approximately every 3-4 days. The EBV positive DLBCL cell line (Farage) was purchased from China Center for Type Culture Collection. All the above cell lines were cultured in RPMI 1640 supplemented with 10% fetal bovine serum (FBS), 1%penicillin and streptomycinthe.

### Flow cytometry analysis of CD45^+^CD19^-^ expression, and fluorescence-activated cell sorting of CD45^+^CD19^-^ cells

To identify the surface markers of LCL, the antibodies conjugated with peridinin chlorophyll complex (Percp), allophycocyanin (APC), phycoerythrin (PE) or fluorescein isothiocyanate (FITC) and included IgG1 isotype controls (Percp, FITC, PE or APC) were used. The cells were labeled with CD45-Percp, CD19-APC, CD20-PE, CD34-PE, CD3-PE, CD16-FITC, CD56-PE, CD14-FITC and were analyzed with flow cytometry (CantoⅡ, BD Biosciences, San Jose, CA, USA). (Information of antibodies was provided in Table S1).

The LCL and Farage cells were incubated with CD45-Percp and CD19-APC and sorted by fluorescence-activated cell sorting (MoFLo, Beckman Coulter, CA, USA).

### Flow cytometry analysis of ALDH expression, and fluorescence-activated cell sorting of ALDH^high^ cells

ALDH activity was measured using the ALDEFLUOR kit per protocol (Stem cell Technologies, Vancouver, BC, Canada). Cells were analyzed and sorted by flow cytometry (AriaⅡ, BD Biosciences, San Jose, CA, USA). All FACS data were analyzed by the Flowjo software (Tree Star, Ashland, OR, USA).

### Clonal analyses in vitro

The CytoSelect 96-Well Cell Transformation Assay (Cell Biolabs, San Diego, CA, USA) was used to assess colony formation. 2,500 and 1,250 cells per well were seeded in soft agar in the flat-bottomed 96-well culture dishes. After 14 days of incubation at 37°C in 5% CO2, soft agar was solubilized. Then cells were incubated with the CyQUANT GR Dye. Colony formation was quantified by the Synergy HT plate reader and the Gen5 software (BioTek, Shoreline, WA, USA). Three independent experiments were performed.

### Expression of stemness genes by western blotting (WB) analysis

The cell lysates were prepared with ice-cold RIPA buffer and centrifuged (12000g for 15 min at 4℃). The protein concentration was determined by BCA assay. 30μg protein sample was electrophoresed on a 10% SDS polyacrylamide gel and electroblotted onto polyvinylidene fluoride membranes. Membranes were incubated in 5% non-fat milk 2 hours and then incubated with primary antibodies, including SOX2, OCT4, Nanog, ABCG2 and β-actin (Zsbio, Beijing, China) at 4°C overnight. After washing with Tris-buffered saline Tween 20 buffer, the membranes were incubated with secondary antibodies conjugated by horseradish peroxidase (HRP) for 2 hours at room temperature. Enhanced chemiluminescence reagent (HaiGene, Harbin, Heilongjiang, China) were used to visualize proteins. (Information of antibodies was provided in Table S1).

### Expression of stemness genes by reverse transcription-PCR (RT-PCR) analysis

Total RNA was obtained using Trizol reagent (Invitrogen, Carlsbad, CA, USA) from the ALDH^high^ and ALDH^-^ Farage cells isolated by FACS. One-step RT-PCR kit (Qiagen, Valencia, CA, USA) was done for RT-PCR on the basis of the manufacturer's protocol. Glyceraldehyde-3-phosphate dehydrogenase (GAPDH) was an internal standard control. The PCR primers sequences for ABCG2, Nanog, OCT4 and SOX2 were listed in the supplementary data (Table S2).

### Xenograft tumor experiments and in vivo tumorigenicity

Immunodeficient NOD/SCID mice (6-8 weeks old) were purchased from Beijing Vital River Laboratory Animal Technology Co., Ltd. These mice were bred in a specific pathogen free (SPF) facility. First, 5×10^6^ unsorted LCL cells were transplanted into NOD/SCID mice by intraperitoneal injection. Then 10^2^, 10^3^, 10^4^, 10^5^, 5×10^5^, and 10^6^ cells of the CD45^+^CD19^+^ and CD45^+^CD19^-^ cells sorted from LCL and Farage cell line were transplanted by intraperitoneal injection into NOD/SCID mice, 10^5^, 5×10^5^, and 10^6^ cell numbers were injected again. 1.5×10^2^, 1.5×10^3^, 1.5×10^4^, and 1.5×10^5^ cells of the ALDH^high^ and ALDH^-^ cells sorted from Farage cell line were also transplanted into NOD/SCID mice by intraperitoneal injection. Mice were kept until about 8 weeks when the mice showed discomfort or distress. All mice were killed at the same time points.

### Immunohistochemistry (IHC) of the xenograft tumors

Xenograft tumors were made paraffin sections. The slides were heated and then deparaffinized. After antigen repair, 1 to 2 drops primary antibodies were added to slides and incubated. Then slides were incubated with 1 to 2 drops HRP-conjugated secondary antibody. Finally, slides were dehydrated, transparent and sealed. (Information of antibodies was provided in Supplementary Table S1).

### Detection of EBV by EBER ISH

The infection of EBV was detected by EBER ISH (EBER DNA Probe, S30172; TRIPLEX, Fujian, China) according to the product manual.

### Statistical analysis

The data were presented as mean±SD. The two-sided student's t test was used to compare the data by statistical software R3.3.2, of which P <0.05 was considered statistically significant.

## Results

### The successful establishment and identification of LCL

**Figure 1:**
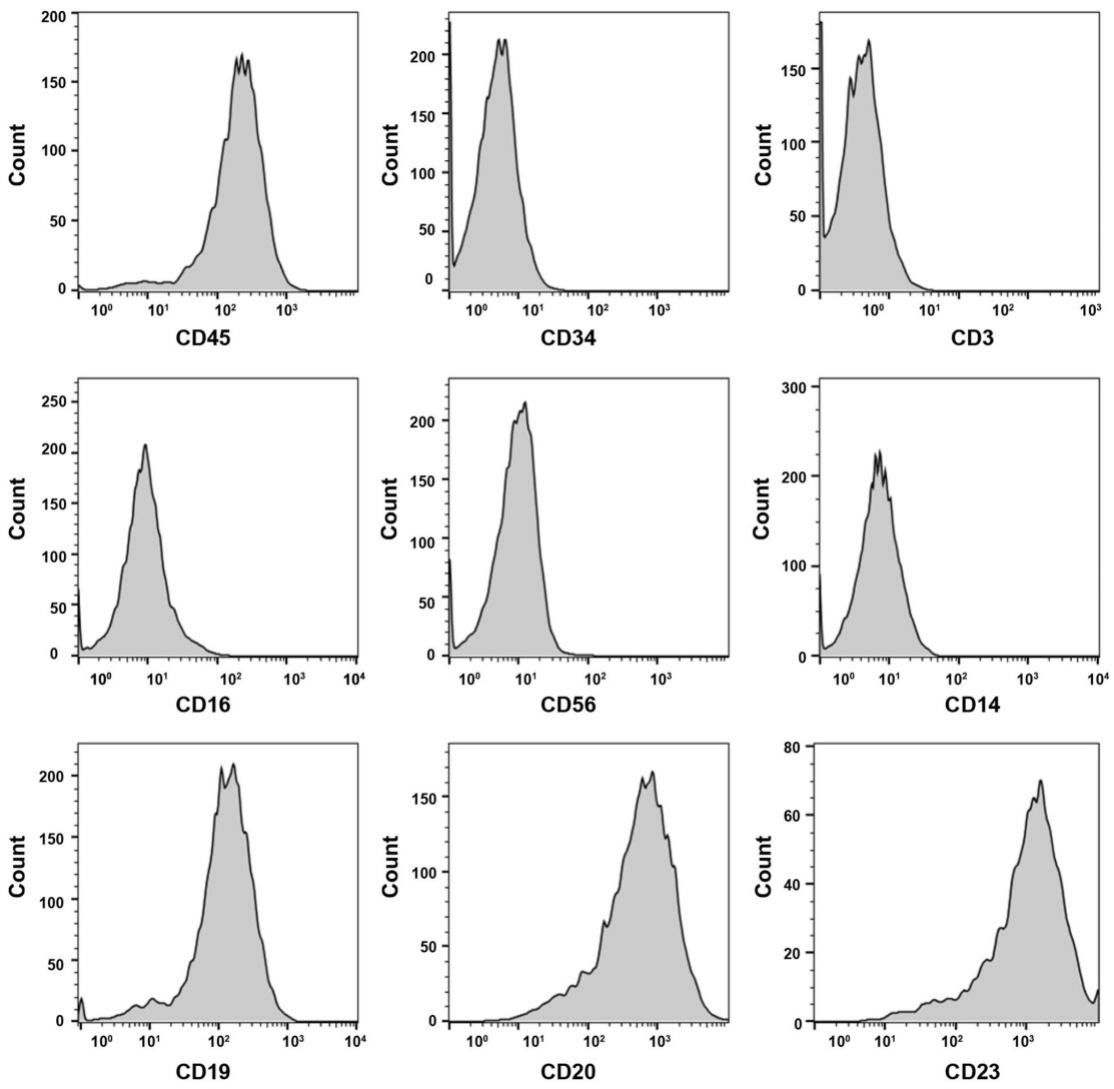
** The surface markers of LCL by flow cytometry analysis.** CD45, CD19, CD20 and CD23 are positive.

About 4 weeks later, LCL was established successfully. Then the LCL was characterized with 8 hematopoietic cell surface markers by FACS analysis. The lymphocyte surface marker of CD45 was positive. The LCL also expressed CD19 and CD20, and two B cell lineage markers, but was negative for the expression of hematopoietic stem cell (CD34), T cell (CD3), NK cell (CD16, CD56) and monocyte cell (CD14) (Fig. 1). The data suggests the LCL is of B cell lineage. To test whether the LCL cell line can be used as a model of lymphoma, the cells were xenotransplanted into NOD/SCID mice. The xenograft tumor had an expression of human CD20, CD19, CD79α, Ki67, Bcl-2, MUM-1 by immunohistochemistry (Supplementary Fig. S1). And the EBV was positive in the xenograft tumor by EBER ISH detection. Two experienced pathologists diagnosed the tumor as a pathological feature of EBV positive DLBCL. It is indicated that the LCL cell line as a model system served to investigate tumorigenesis of DLBCL is possible.

### The existence of CD45^+^CD19^-^ cells population in LCL and Farage cell line

**Figure 2:**
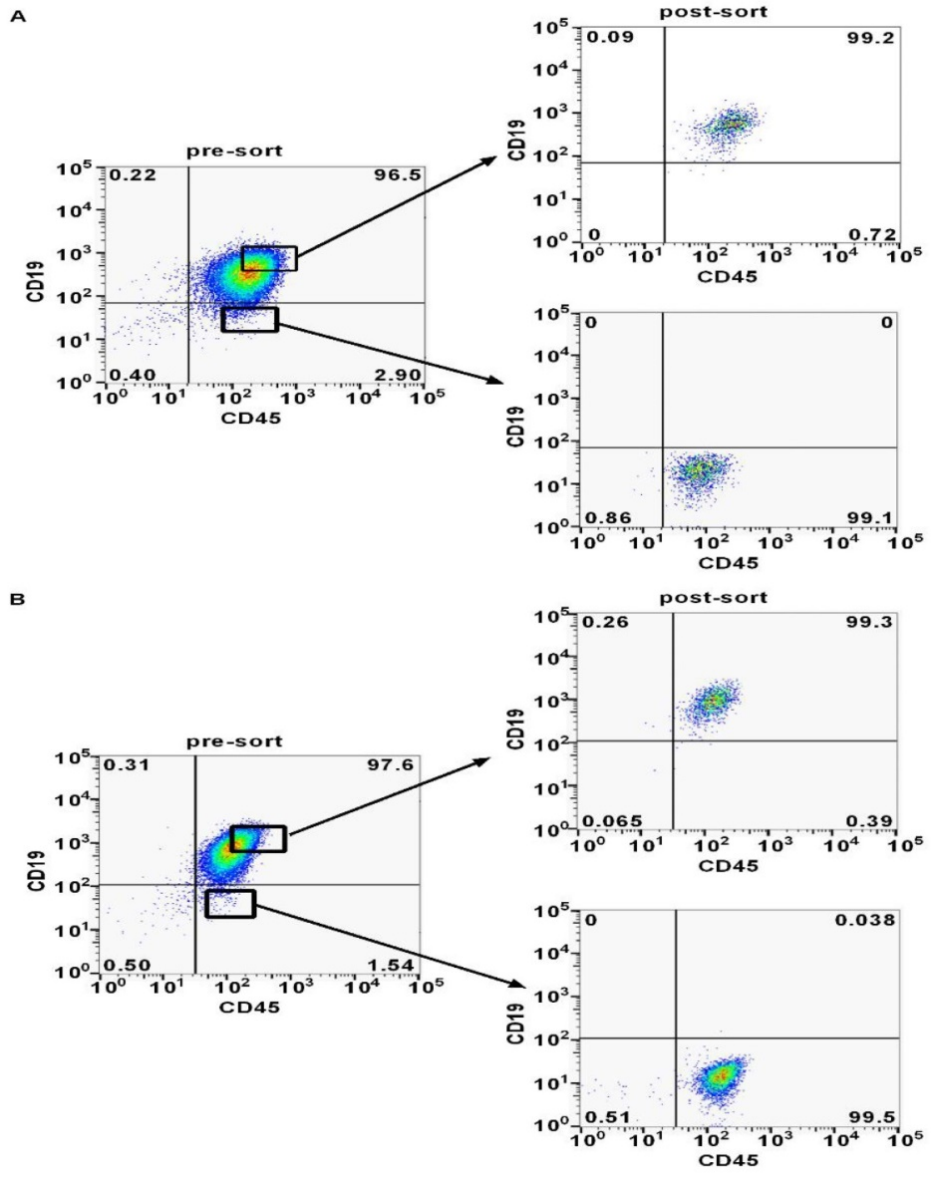
** CD45^+^CD19^+^ and CD45+CD19^-^ cells were analyzed and sorted by flow cytometer. (A)** The proportion of CD45^+^CD19^-^ cells and the post-sort purity in LCL cell line. **(B)** The proportion of CD45^+^CD19^-^ cells and the post-sort purity in Farage cell line.

Previous studies reported that minor CD45^+^CD19^-^ cells may identify CSCs in MCL 13-15. In order to find out whether the CD45^+^CD19^-^ cells were expressed in DLBCL, we analyzed LCL and Farage cells via flow cytometry. The CD45^+^CD19^-^ cells accounted for 1-3% in the two cell lines (Fig. 2). A portion of CD45^+^CD19^-^ cells and CD45^+^CD19^+^ cells were sorted separately for the further studies. Post-sorting analyses showed these two sub-populations had a purity of ≥99% in the desired population (Fig. 2).

### CD45^+^CD19^-^ cells are not more tumorigenic than the CD45^+^CD19^+^ cells

**Figure 3:**
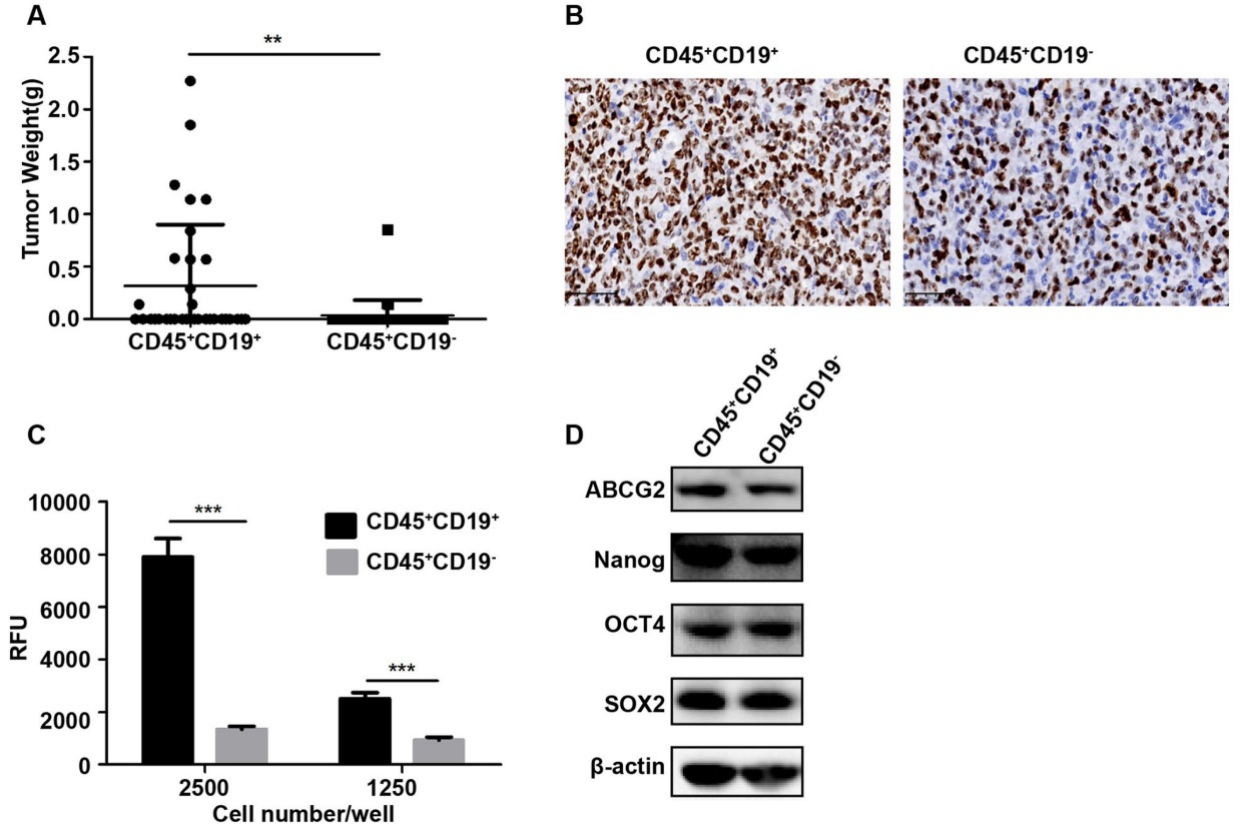
** Characterization of CD45^+^CD19^+^ and CD45^+^CD19^-^ cells in LCL cell line. (A)** the weight of tumors generated by CD45^+^CD19^+^ cells were higher than CD45^+^CD19^-^ cells, *p≤0.05. **(B)** Ki-67 analysis of CD45+CD19+ and CD45+CD19- cells derived tumor xenografts by IHC (×400). **(C)** cells were plated at clone density (2500 cells and 1250 cells per well) and cultured for 14 days, colony formation was quantified using the fluorescent CyQUANT GR Dye. Three independent experiments were performed, ***p≤0.001. **(D)** the expression levels of ABCG2, Nanog, OCT4 and SOX2 were evaluate. β-actin was used as a loading control. There was no difference between the two groups.

In vivo, the identification of putative CSCs in immunodeficient xenograft model is the ultimate proof. To determine whether CD45^+^CD19^-^ could serve as a marker for DLBCL, we also conducted xenotransplantation in NOD/SCID mice. When more than 100,000 cells were transplanted into the mice, both of the two sub-populations generated tumors. Compared with the same number of cells, the CD45^+^CD19^+^ population generated more tumors (Table 1) and larger tumors (p<0.05, Fig.3A). However, with a small number of cells (100 to10, 000) inoculated, two sub-populations did not generate a tumor (Table 1). Ki-67, the proliferative antigen, showed higher expression in CD45^+^CD19^+^ xenograft tumor (Fig. 3B), which was consistent with the larger tumors in this sub-population.

**Figure 4:**
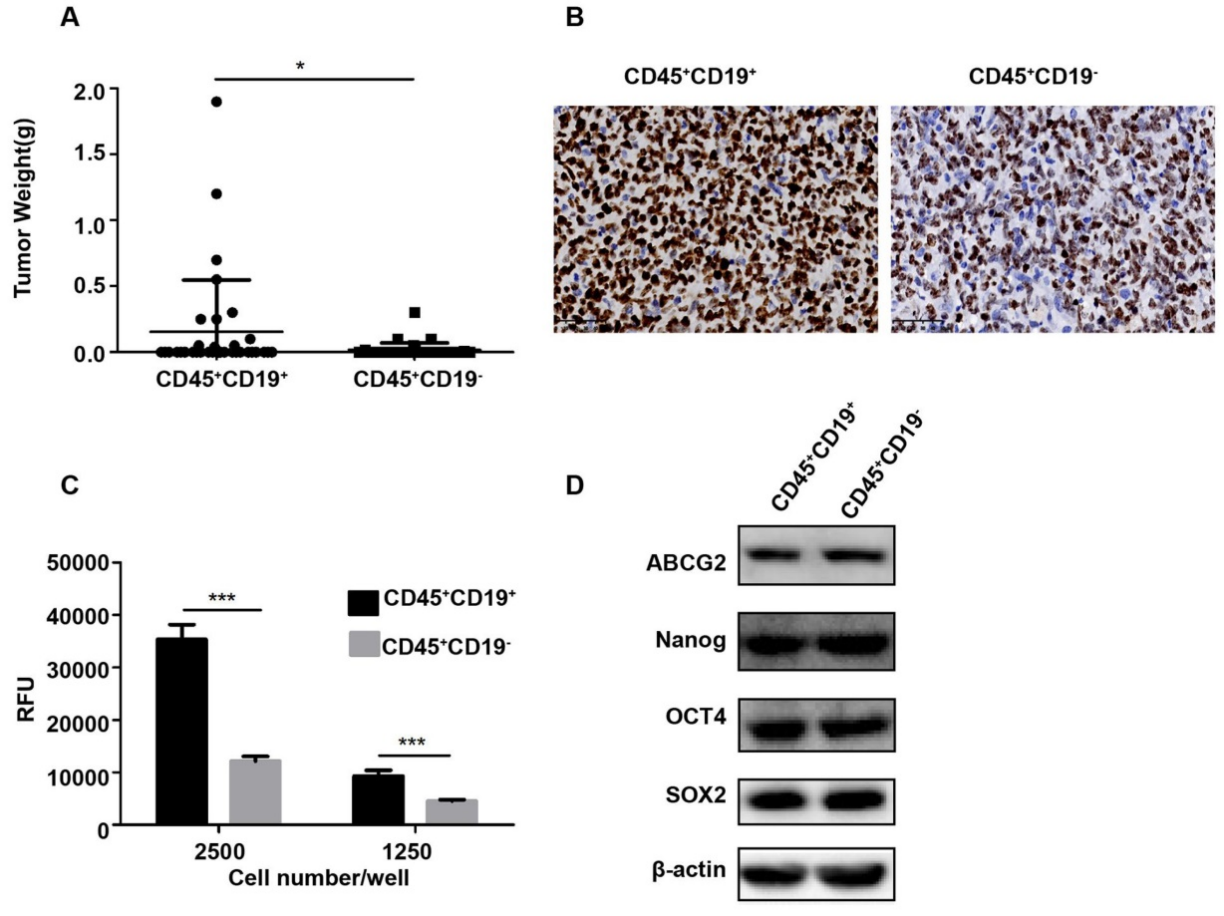
** Characterization of CD45^+^CD19^+^ and CD45^+^CD19^-^ cells in Farage cell line. (A)** the weight of tumors generated by CD45^+^CD19^+^ cells were higher than CD45^+^CD19^-^ cells, **p≤0.01. **(B)** Ki-67 analysis of CD45^+^CD19^+^ and CD45^+^CD19^-^ cells derived tumor xenografts by IHC (×400). **(C)** cells were plated at clone density (2500 cells and 1250 cells per well) and cultured for 14 days, colony formation was quantified using the fluorescent CyQUANT GR Dye. Three independent experiments were performed. ***p≤0.001. **(D)** the expression levels of ABCG2, Nanog, Oct4 and SOX2 were evaluated. β-actin was used as a loading control. There was no difference between the two groups.

Then, CD45^+^CD19^+^ and CD45^+^CD19^-^ cells were isolated from Farage cell line and similar results were observed. When 100,000 CD45^+^CD19^+^ cells were injected into the mice, the tumor incidence was 33.33% (two of six; Table 1). However, CD45^+^CD19^-^ cells did not give rise to any tumor (zero of six; Table 1) in the presence of the same number of these cells. With 1, 000, 000 CD45^+^CD19^+^ cells injected, we found a tumor incidence of 100% (six of six; Table 1), compared with 66.67% (four of six; Table 1) in CD45^+^CD19^-^ cells. And tumors formed by CD45^+^CD19^+^ cells were larger than those formed by CD45^+^CD19^-^ cells (p<0.05, Fig. 4A). Immunohistochemical analysis showed increased Ki-67 expression in the CD45^+^CD19^+^ derived tumors (Fig. 4B).

To confirm tumorigenic activities were not enriched in the CD45^+^CD19^-^ cells, we repeated tumor xenotransplantation experiments using purified CD45^+^CD19^+^ cells and CD45^+^CD19^-^ cells from LCL and Farage and observed similar results (Table S3; Fig. S2).

These results show that both CD45^+^CD19^+^ and CD45^+^CD19^-^ cells possess the tumor-initiating capacity at a certain number of cells, while CD45^+^CD19^-^ cells are not more tumorigenic than the CD45^+^CD19^+^ cells.

### CD45^+^CD19^+^ cells have stronger clonogenicity than CD45^+^CD19^-^ cells

We further tested the colony formation ability in vitro, which partially evaluates the tumorigenicity of the cells in vitro 25, 26. Purified CD45^+^CD19^+^ and CD45^+^CD19^-^ cells from LCL and Farage were used. As a result, CD45^+^CD19^+^ cells showed greater colony formation than CD45^+^CD19^-^ cells (Fig. 3C, 4C), which was in line with xenotransplantation results in vivo.

### The expression of stemness genes is similar between CD45^+^CD19^+^ cells and CD45^+^CD19^-^ cells

The expression of stemness genes is also used to identify CSCs 27-30. OCT4, SOX2, Nanog and ABCG2 are considered as important factors in the maintenance of stem cells. So, the expression of OCT4, SOX2, Nanog and ABCG2 were detected by WB analyses in our study. The result showed there was no difference between the CD45^+^CD19^+^ and CD45^+^CD19^-^ cells in LCL and Farage cell line (Fig. 3D, 4D).

### ALDH^high^ cells are more tumorigenic than the ALDH^-^ cells

**Figure 5:**
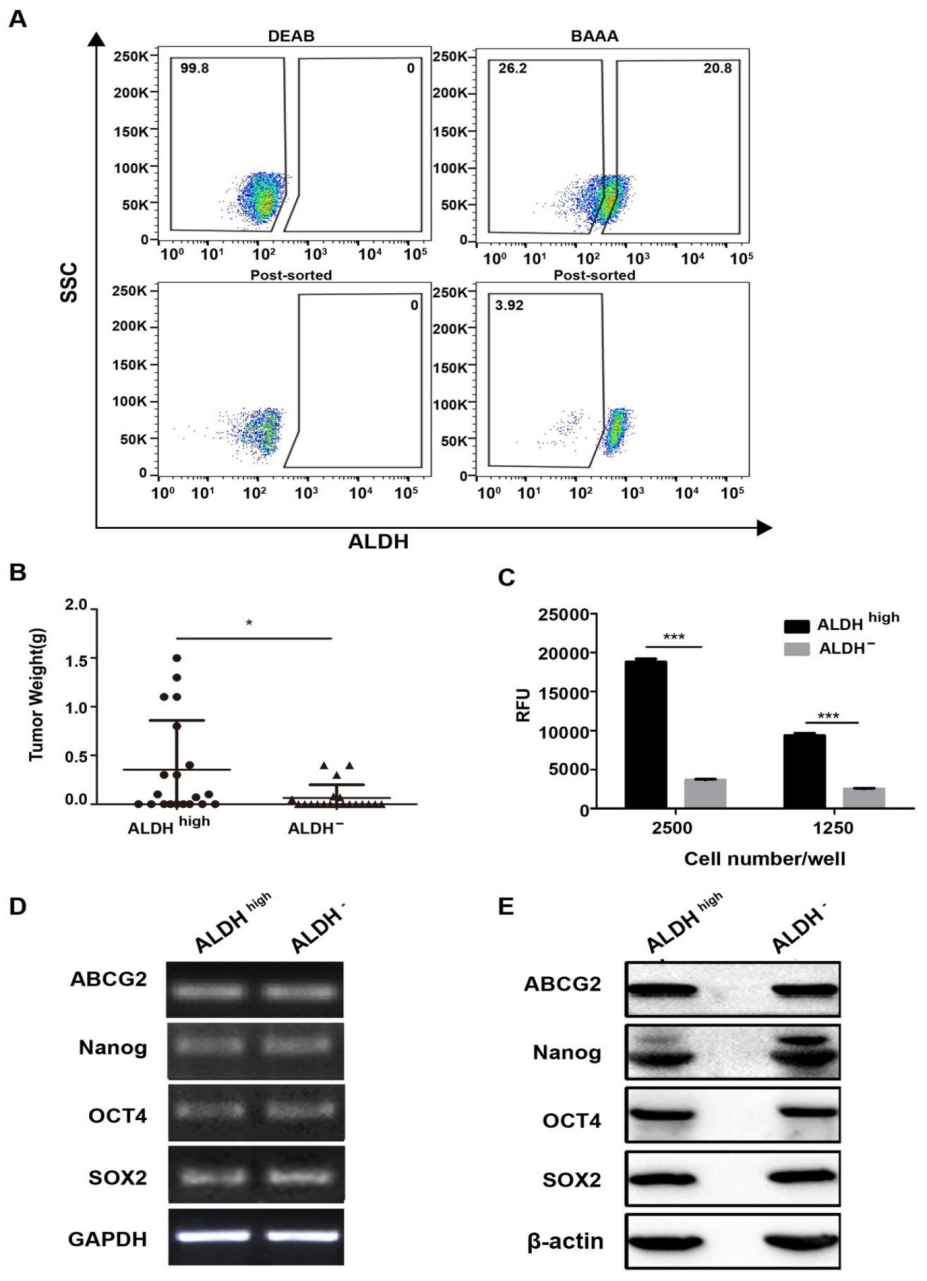
** Characterization of ALDHhigh and ALDH- cells in Farage cell line. (A)** The proportion of ALDH^high^ cells and the post-sort purity in Farage cell line. **(B)** the weight of tumors generated by ALDH^high^ cells were higher than ALDH- cells. *p≤0.05. **(C)** cells were plated at clone density (2500 cells and 1250 cells per well) and cultured for 14 days, colony formation was quantified using the fluorescent CyQUANT GR Dye. Three independent experiments were performed. ***p≤0.001. **(D)** and **(E)** the expression levels of ABCG2, Nanog, Oct4 and SOX2 were evaluated by RT-PCR and WB analysis. GAPDH and β-actin were used as loading controls respectively. There was no difference between the two groups.

Our study indicated CD45^+^CD19^-^ surface marker cannot identify CSCs populations in DLBCL. So, we further explored the ALDH activity which had been reported as a potential marker of CSCs in many tumors 31-33 in Farage cell line. In order to obtain ALDH^high^ cells, only about 10% of the most brightly stained cells were selected (Fig. 5A). After 1,500 ALDH^high^ cells were injected into the NOD/SCID mice, these cells gave rise to tumors (40% incidence, two of five; Table 2). With 15,000 ALDH^high^ cells injected, tumor incidence was 80% (four of five; Table 2). With 150,000 ALDH^high^ cells injected, all five mice generated tumors (Table 2). In contrast, no tumor was generated with 1,500 ALDH^-^ cells (zero of five; Table 2). Tumor did arise when 15,000 ALDH^-^ cells were injected (two of five; Table 2). Although ALDH^-^ cells generated tumors, the tumors derived by ALDH^high^ cells were larger than those induced by ALDH^-^ cells (Fig. 5B). These results showed that CSCs were enriched in the ALDH^high^ cells.

### ALDH^high^ cells have stronger clonogenicity than ALDH^-^ cells

The colony formation of ALDH^high^ and ALDH^-^ in Farage cell line was also identified. In accordance with the tumorigenicity, the ALDH^high^ cells had stronger colony formation ability than ALDH^-^ cells. (Fig. 5C).

### The expression of stemness genes is similar between ALDH^high^ and ALDH^-^ cells

However, the expression levels of stemness genes did not show any significant difference between the ALDH^high^ and ALDH^-^ cells (Fig. 5D, 5E). Most probably, although the ALDH^high^ cells enrich CSCs, its purity is not high enough. Therefore, it is difficult to obtain the difference between the two subgroups.

## Discussion

CSCs, as defined by the American Association for Cancer Research Workshop denotes cells within a tumor that possess the capacity to self-renewal and to differentiate into the heterogeneous lineages of cancer cells that constitute the tumor 34. The CSCs theory suggests that tumor growth is drived by a small number of CSCs hidden in cancers. It is helpful to explain some clinical phenomenon, such as recurrence after initially successful chemotherapy and/or radiotherapy, tumor dormancy, and metastasis. CSCs were identified in many common types of cancer, including leukemia, breast cancer, brain cancer, colorectal cancer and so on. The CSCs theory has stimulated therapeutic strategies for these tumors, not aimed at eliminating tumor bulk, but rather at eradicating CSCs, the cell which maintains tumor growth 35. Isolation of this cell subpopulation by special markers is an important step in identifying these properties. Although CD34^+^CD38^-^ 6, 7, CD133^+^ 8 and CD44^+^CD24^-^ 9-11 have been widely used as CSCs markers in human acute myeloid leukemia, brain tumor and breast cancer respectively. However, there are still no distinct markers of CSCs in many cancer subtypes, including lymphoma.

NHL is a pathologic type of B cell lymphoma, including DLBCL, chronic lymphocytic leukemia/small lymphocytic lymphoma (CLL/SLL), follicular lymphoma (FL), MCL, and so on. Among these types of tumor, CD45^+^CD19^-^ has been identified as a potential marker of CSCs in MCL 13-15. So, we also explored the possibility of CD45^+^CD19^-^ as a potential marker of DLBCL CSCs in vivo and in vitro. However, both of CD45^+^CD19^-^ cells and CD45^+^CD19^+^ cells did not generate tumors until more than 100,000 cells inoculated in NOD/SCID mice, even CD45^+^CD19^+^ cells generated more and larger tumors, as well as the soft agar colony formation in vitro. As for CSCs, one important ability is to form tumors at low cell numbers. In theory, injection of a single CSC might generate tumor in xenotransplantation mouse model 36. Most tests on CSCs-induced tumorigenesis have used 100∼1,000 cells as the smallest number of cells injected 37-42. Therefore, CD45^+^CD19^-^ may not be suitable as a marker of CSCs in DLBCL. Only 100 CD45^+^CD19^-^ cells isolated from the PBMCs of MCL patients generated tumors 13, however, CD45^+^CD19^-^ cells sorted from the DLBCL cell lines did not generate tumors at the low cell densities in our study. The possible reasons are as follows. Firstly, the different pathogenesis of MCL and DLBCL; Secondly, the role of EBV. Our cell lines are EBV positive, whereas all MCL patients are EBV negative; Thirdly, the role of the microenvironment. The CD45^+^CD19^-^ cells were isolated from the PBMCs of MCL patients who were in the leukemic phase. As disease progresses, the change of the microenvironment might cause the different markers of CSCs. Moreover, both CD45^+^CD19^-^ cells and CD45^+^CD19^+^ cells sorted from the DLBCL cell lines generated tumors with enough number of cells injected in our study. This was consistent with the previous study in MCL, which the CD45^+^CD19^+^ cells isolated from the MCL cell lines also generated tumors with 5×10^5^ and 10^6^ cells injected 13. We performed the experiments twice by using LCL and Farage cell lines and obtained similar results. These results suggest not only CD45^+^CD19^-^ but also CD45^+^CD19^+^ cells isolated from the cell lines can drive tumors as long as enough cells are transplanted in mice. This phenomenon may be explained by the “clonal model”, which suggests that each cell within a tumor has equal capacity to generate new tumors 43. So CD45^+^CD19^-^ is not suitable as a marker of CSCs in DLBCL, how about the ALDH activity?

High ALDH activity has been demonstrated in CSCs of many tumor types, including MCL 44 and Burkitt lymphoma 45. However, it has not been reported as a marker of CSCs in DLBCL, although ALDH1 expression was immunohistochemically examined in DLBCL in the previous study 22-24. In our study, ALDHhigh cells were sorted using Aldefluor assay kit (StemCell Technologies, Durham, NC, USA) by flow cytometry and identified as a marker of CSCs in DLBCL. The results revealed 1,500 ALDH^high^ cells generated tumors while same number of ALDH^-^ cells did not. ALDH^high^ cells generated more and larger tumors than ALDH^-^ cells when both of two subgroups generated tumors with more cells injected. The similar result was obtained in vitro clonogenicity experiments. These data suggest ALDH activity identifies a population of DLBCL cells enriched for CSCs activity. However, 150 ALDH^high^ cells cannot induce tumors in our study. Maybe ALDH^high^ population is still heterogeneous, which consists of subsets of cells with different tumorigenic potential. Certainly, ALDH^high^ cells combining with definitive CSCs surface markers may further enrich CSCs population in other studies, such as hepatocellular carcinoma, head and neck carcinoma and ovarian cancer 17, 21, 32. Nevertheless, no specific surface markers have been found in DLBCL nor did we identify CSCs by combining ALDH activity with surface markers in DLBCL.

Expression of stemness genes is also used to identify CSCs. OCT4, Sox2, and Nanog are transcription factors that are analyzed in most studies. They are often expressed in pluripotent embryonic stem cells, germ cells, certain committed progenitors and cancer cells 35. ABCG2 is one of the major mediators in the ABC family of transporter proteins, which has been also used to identify both normal cells and CSCs 46. In our study, we found these genes were similarly expressed in CD45^+^CD19^+^ cells, CD45^+^CD19^-^ cells, ALDH^-^ cells and ALDH^high^ cells. As mentioned above, CSCs are not enriched in CD45^+^CD19^-^ cells and CD45^+^CD19^+^ cells, so we can understand the similar expression of these genes. To our surprise, this phenomenon even happened in ALDH^-^ cells and ALDH^high^ cells, which maybe because the purity of enriched CSCs in ALDH^high^ cells is not high enough or other unknown related genes exist. So the expression of stemness genes regulated ALDHhigh cells will be performed by gene expression profile or gene sequencing in order to obtain sufficient verifications of ALDHhigh cells in our future work. On the other hand, perhaps their expression is a feature of malignant transformation and not exclusive to CSCs 36.

Above all, CSCs are not enriched in the CD45^+^CD19^-^ cells but in the ALDH^high^ cells of DLBCL cell lines although the tumorigenic ability of ALDH^high^ cells is limited because its purity is not high enough. ALDH activity may be used as a marker of CSCs in DLBCL, which will be helpful for developing the prognosis and therapeutic strategies. We also hope to provide an early warning indicator for monitoring the occurrence of lymphoma in patients with EBV infection by regularly detecting the expression of ALDH activity, since EBV play an important role in the pathogenesis of DLBCL. And the stemness and tumorigenic properties of ALDHhigh cells in EBV negative DLBCL also deserve further exploration.

## Supplementary Material

Supplementary figures and tables.

## Figures and Tables

**Table 1:** Tumor-initiating capacity of limiting dilutions of CD45^+^CD19^+^ and CD45^+^CD19^-^cells from the LCL and Farage cell lines

Cell	LCL cell line		Farage cell line	
Cell type	CD45^+^CD19^+^	CD45^+^CD19^-^	CD45^+^CD19^+^	CD45^+^CD19^-^
No. of cells injected	Tumors formed		Tumors formed	
10^6^	5/6	2/6	6/6	4/6
5×10^5^	4/5	0/6	3/5	3/6
10^5^	3/6	1/6	2/6	0/6
10^4^	0/6	0/6	0/6	0/6
10^3^	0/6	0/6	0/6	0/6
10^2^	0/6	0/6	0/6	0/6

**Table 2:** Tumor-initiating capacity of limiting dilutions of ALDH^high^ and ALDH^-^ cells from the Farage cell line

Cell	Farage cell line	
Cell type	ALDH^high^	ALDH^-^
No. of cells injected	Tumors formed	
1.5×10^5^	5/5	4/5
1.5×10^4^	4/5	2/5
1.5×10^3^	2/5	0/5
1.5×10^2^	0/5	0/5

## Abbreviations

CSCs: cancer stem cells
DLBCL: diffuse large B cell lymphoma
MCL: mantle cell lymphoma
ALDH: aldehyde dehydrogenase
NHL: non-Hodgkin's lymphoma
EBV: Epstein-Barr virus
LCL: lymphoblastoid cell line
SP: side population
PBMC: peripheral blood mononuclear cells
FBS: fetal bovine serum
Percp: peridinin chlorophyll complex
APC: allophycocyanin
PE: phycoerythrin
FITC: fluorescein isothiocyanate
WB: western blotting
RT-PCR: reverse transcription-PCR
GAPDH: Glyceraldehyde-3-phosphate dehydrogenase
IHC: Immunohistochemistry
SPF: specific pathogen free
CLL/SLL: chronic lymphocytic leukemia/small lymphocytic lymphoma
FL: follicular lymphoma
