# Supplementary material for: Cancer Stem Cells of Diffuse Large B Cell Lymphoma Are Not Enriched in the CD45+CD19- cells but in the ALDHhigh Cells
Source: J Cancer. 2020 Jan 1;11(1):142–52. doi: 10.7150/jca.35000 (PMC6930399; doi:10.7150/jca.35000)
Supplement: Supplementary file 1 — Supplementary figures and tables. [file jcav11p0142s1.pdf]

Table S1

Antibody list

Anti-human	Conjugation	Source	Catalog number	Clone	Application
Anti-human CD45	Percp	BD	347464	2D1	flow cytometry
Anti-human CD19	APC	BD	340437	SJ25-C1	flow cytometry
Anti-human CD20	PE	BD	346595	L27	flow cytometry
Anti-human CD3	PE	BD	347347	SK7	flow cytometry
Anti-human CD14	FITC	BD	347493	MφP-9	flow cytometry
Anti-human CD16	FITC	BD	347523	NKP15	flow cytometry
Anti-human CD56	PE	BD	347747	MY31	flow cytometry
Anti-human CD34	PE	BD	348057	8G12	flow cytometry
Anti-ABCG2	none	Abcam	ab207732	EPR20080	WB
Anti-Nanog	none	Abcam	ab109250	EPR2027(2)	WB
Anti-OCT4	none	Abcam	ab19857	polyclonal	WB
Anti-SOX2	none	Wanlei	WL00982	polyclonal	WB
Mouse Anti-β actin	none	Zsbio	TA-09	OTI1	WB
Goat Anti-Mouse IgG	HRP	ABclonal	AS003	polyclonal	WB
Goat Anti-Rabbit IgG	HRP	ABclonal	AS014	polyclonal	WB
Anti-human CD45	none	Maixin	Kit-0024	PD7/26+2B11	IHC
Anti-human CD3	none	Maixin	Kit-0003	SP7	IHC
Anti-human CD19	none	Maixin	MAB-0646	LE-CD19	IHC
Anti-human MUM1	none	Maixin	MAB-0573	MUM1p	IHC
Anti-human Bcl-6	none	Maixin	MAB-0598	LN22	IHC
Anti-human CD10	none	Gene Tech	GT200407	56C6	IHC
Anti-human CD20	none	Gene Tech	GM075507	L26	IHC
Anti-human CD79α	none	Gene Tech	GM705007	JCB117	IHC
Anti-human Bcl-2	none	Gene Tech	GM088704	124	IHC
Anti-human cyclin-D1	none	Gene Tech	GT205807	SP4	IHC
Anti-human ki67	none	Gene Tech	GT210107	SP6	IHC
Goat Anti-Mouse/ Rabbit IgG	HRP	Zsbio	PV-6000	polyclonal	IHC

Table S2

Primer sequences list.

Gene	Forward primer5'-3'	Reverse primer5'-3'
ABCG2	ACGAACGGATTAACAGGGTCA	CTCCAGACACACCACGGAT
Nanog	AATACCTCAGCCT CCAGCAGAT	TGCGTCACACCA TTGCTATTCTTC
OCT4	CTTGCTGCAGAAGTGGGTGGAGGAA	CTGCAGTGTGGGTTTCGGGCA
SOX2	AAATGGGAGGGGTGCAAAAGAG GAG	CAGCTGTCATTTGCTGTGGGTGATG
GAPDH	ACCACA GTCCATGC CATCAC	TCC ACCACCCTGTTGCTGTA

Table S3

Tumor-initiating capacity of limiting dilutions of $CD45^+CD19^+$ and $CD45^+CD19^-$cells from the LCL and Farage cell lines again.

Cell	LCL cell line		Farage cell line	
Cell type	$CD45^+CD19^+$	$CD45^+CD19^-$	$CD45^+CD19^+$	$CD45^+CD19^-$
No. of cells injected	Tumors formed		Tumors formed	
10^6	6/6	3/6	6/6	4/6
$5×10^5$	4/6	3/6	6/6	5/6
10^5	4/6	3/6	3/6	0/6

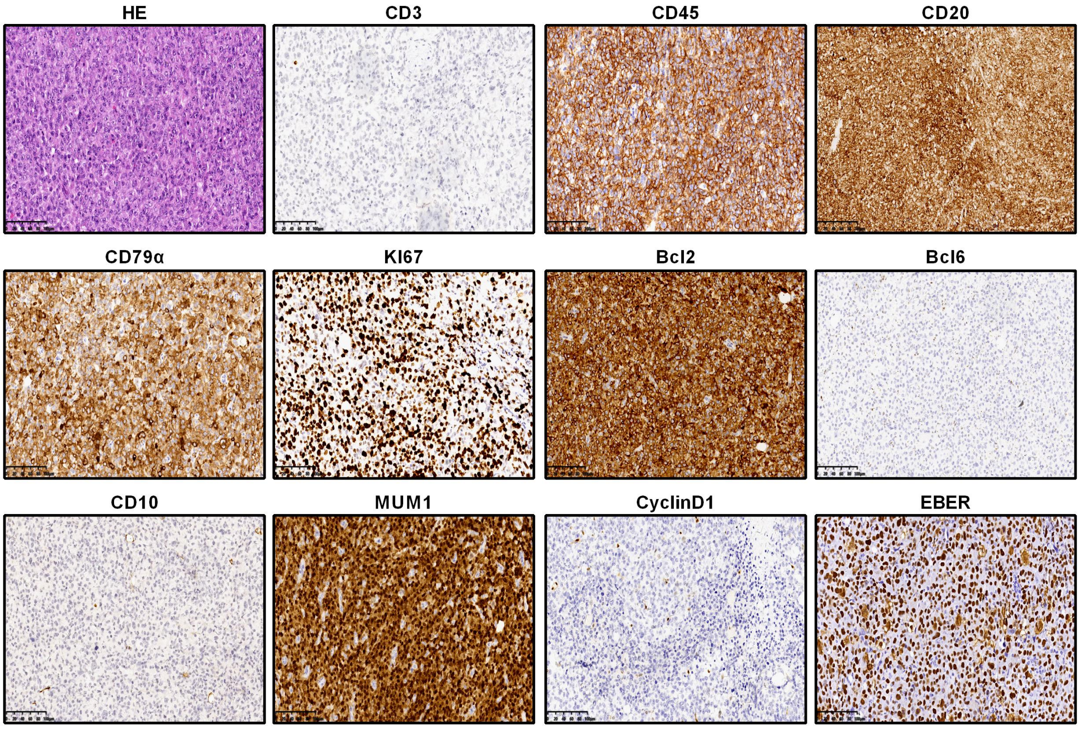

Figure S1. Pathological features of LCL xenotransplantation tumor (×200). They are consistent with the pathological features of diffuse large B cell lymphoma.

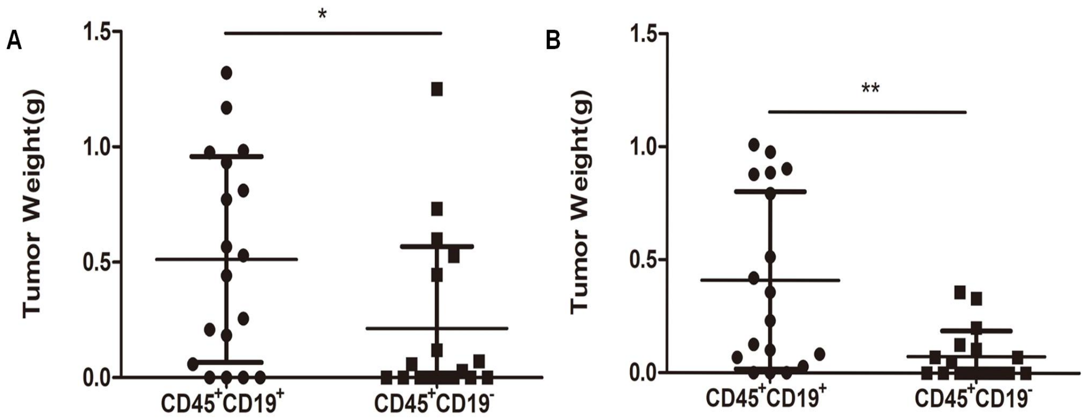

Figure S2.

Verify tumorgenicity of CD45⁺CD19⁺ and CD45⁺CD19⁻ cells in LCL and Farage cell line again. (A) the weight of tumors generated by $CD45^+CD19^+$ cells were higher than $CD45^+CD19^-$ cells from the LCL cell line. (B) the weight of tumors generated by $CD45^+CD19^+$ cells were higher than $CD45^+CD19^-$ cells from the Farage cell line. *$p \leqslant 0.05$, **$p \leqslant 0.01$.
